# Can direct smear results that are routinely collected at health centre level be used for monitoring the impact of mass drug administration with praziquantel on schistosomiasis in Burundi? A preliminary assessment

**DOI:** 10.1186/s13071-020-04076-4

**Published:** 2020-04-21

**Authors:** Paul Bizimana, Katja Polman, Giuseppina Ortu, Meryam Krit, Frédéric Nsabiyumva, Audace Nkeshimana, Urlich Bijabuka, Marcelline Nibakire, Jean-Pierre Van Geertruyden

**Affiliations:** 1 Global Health Institute, Department of Epidemiology and Social Medicine, Faculty of Medicine and Health Sciences, University of Antwerp, Antwerp, Belgium; 2 Département des Sciences de la Santé Publique, Direction de la Formation, Institut National de Santé Publique, Bujumbura, Burundi; 3 Département de Médecine Communautaire, Faculté de Médecine de Bujumbura, Université du Burundi, Bujumbura, Burundi; 4 Département des Sciences de la Santé Publique, Institut Universitaire des Sciences de la Santé et de Développement Communautaire, Bujumbura, Burundi; 5 Faculté de Médecine, Université de Ngozi, Ngozi, Burundi; 6 Medical Helminthology Unit, Department of Biomedical Sciences, Institute of Tropical Medicine, Antwerp, Belgium; 7 Section of Infectious Diseases, Department of Health Sciences, VU Amsterdam, Amsterdam, The Netherlands; 8 Global Health Consultant, London, UK; 9 Biostatistics and Epidemiology Unit, Department of Biomedical Sciences, Institute of Tropical Medicine, Antwerp, Belgium; 10 Département de Médecine Interne, Faculté de Médecine de Bujumbura, Université du Burundi, Bujumbura, Burundi; 11 Bureau de la Municipalité Sanitaire de Bujumbura, Ministère de la Santé Publique et de la Lutte contre le Sida, Bujumbura, Burundi

**Keywords:** Burundi, Direct smear, Health centre, Mass drug administration, Monitoring, Routine data, Praziquantel, Schistosomiasis

## Abstract

**Background:**

Intestinal schistosomiasis is still a public health problem in Burundi. Since 2008, annual mass drug administration with praziquantel has been rolled out in 11 endemic districts. The national programme relies on school-based surveys with kato-katz to monitor the impact of mass drug administration. We explored whether routine data on intestinal schistosomiasis as determined by direct fecal smears at health centre level could be used.

**Methods:**

From the Burundian National Health Information System, we collected routine incidence data on intestinal schistosomiasis as determined by direct smear examination in all 45 sanitary districts during 2011–2015. A temporal trends analysis was performed using a mixed negative binomial regression. Sanitary districts with mass drug administration campaigns with praziquantel (*n* = 11) were compared with those without (*n* = 34). In addition, prevalence data on intestinal schistosomiasis based on kato-katz results from a school-based national mapping in 2014 were compared with the incidence data in health centres based on direct smear results, in the same 45 sanitary districts.

**Results:**

In the 11 sanitary districts applying mass drug administration with praziquantel, the incidence rate decreased significantly for the years 2014 (*β*_2014_ = − 0.826, *P* = 0.010) and 2015 (*β*_2015_ = − 1.294, *P* < 0.001) and for the five-year period (*β* = − 0.286, *P* < 0.001), whereas in the 34 districts where mass drug administration was not delivered, there was no significant decrease over time (*β* = − 0.087, *P* = 0.219). In most of the 45 sanitary districts, the low prevalence based on kato-katz in school children was confirmed by low incidence rates based on direct smears in the health centres.

**Conclusions:**

National Health Information System surveillance data, based on routinely collected direct smear results at health centre level, may be able to monitor the impact of mass drug administration with praziquantel on intestinal schistosomiasis in Burundi. Control and elimination of intestinal schistosomiasis call for integration of adequate diagnosis and treatment into routine activities of primary health care facilities, as recommended by the World Health Organization since more than 20 years. When moving towards elimination, more sensitive tests, such as the point-of-care circulating cathodic antigen assay are desirable.
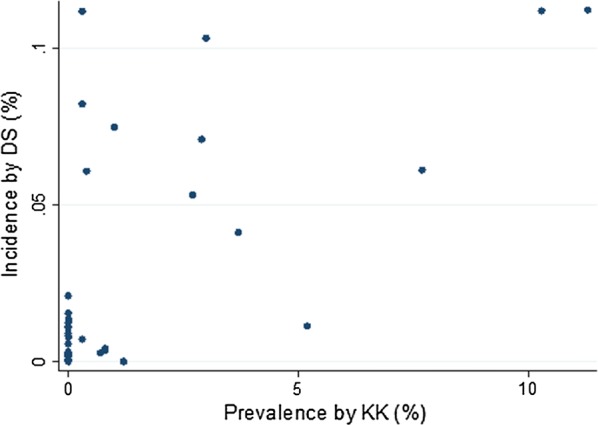

## Background

Schistosomiasis is a parasitic disease caused by the genus *Schistosoma* [1, 2]. It is one of the neglected tropical diseases (NTD). Linked to poverty, schistosomiasis is more prevalent in countries and regions where hygienic conditions are precarious and access to safe water and adequate sanitation is limited [2]. Between 200–250 million of people in the world are infected [3] and 600–780 million are at risk [3, 4]. The sub-Saharan African countries are the most affected by this disease, with more than 90% of the total burden [5, 6].

In Burundi, intestinal schistosomiasis has been a public health challenge for many decades [7, 8]. According to a nationwide school-based survey performed in 2007, based on the kato-katz (KK) test [9], 11 out of the 45 sanitary districts (SD) in Burundi were considered at risk of intestinal schistosomiasis. Since 2008, these 11 SD have been targeted for annual mass drug administration (MDA) with praziquantel (PZQ) to school-aged children between 5 and 15 years-old.

To measure the impact of MDA, epidemiological surveys using KK test were performed in a number of primary schools, chosen as sentinel sites [10]. These surveys demonstrated a decrease in *S. mansoni* infection from 12.7% in 2007 (baseline) to 1.1% in 2011 [10].

After six years of annual MDA in the targeted areas, a school-based national mapping was implemented to reassess the extent of the schistosomiasis problem and to determine whether it was possible to move to the elimination phase [10]. The results from the KK test showed that there was a decline in the prevalence of infection, from 12.7% in 2007 (baseline) to 2.2% in 2014 [10].

Although the epidemiological surveys in sentinel sites and the national mapping provide a good estimation of the prevalence of schistosomiasis, it is a very laborious and costly undertaking [11, 12] and often requires external financial support, especially in resource-limited countries such as Burundi [9, 10]. It is therefore important to look for other cost-effective and sustainable strategies to monitor the impact of MDA campaigns.

In this paper, we aim to evaluate whether routine data on intestinal schistosomiasis as determined by direct smear (DS) stool examination and reported by health centres (HC) to the Directorate of National Health Information System (DNHIS), could be used as an approach to monitor the impact of MDA on intestinal schistosomiasis in Burundi.

## Methods

### Data on the study area and population

Burundi is an East African country. It is limited (i) in the north by Kanyaru River and Cohoha Lake, which separate it from Rwanda, (ii) in the east and southeast by Malagarazi River and Tanganyika Lake, respectively, which separate it from Tanzania, and (iii) in the west and northwest by Tanganyika Lake and Rusizi River, respectively, which separate it from Democratic Republic of Congo.

From 2011 to 2015 (the period of this study), Burundi had 17 sanitary provinces (SP) and 45 SD. The number of HC increased from 284 in 2011 to 355 in 2015 in the 11 SD considered at risk of intestinal schistosomiasis, and from 528 in 2011 to 643 in 2015 in the remaining 34 SD.

All the 11 SD considered at risk of intestinal schistosomiasis, and targeted by MDA campaigns with PZQ, are located near great rivers (the Kanyaru and Rusizi rivers in the north and northwest, respectively) and lakes (Lake Cohoha in the north and Lake Tanganyika in the west, south and southeast), which separate Burundi from neighbouring countries. This is not the case for the 34 remaining SD, which are mainly located far from the borders.

The average population per SD was 192,166 in 2011 and 211,298 in 2015. The average population density was 311 per km^2^ in 2011 and 342 per km^2^ in 2015.

### Functioning of the National Health Information System

The Burundian National Health Information System (NHIS) consists of three levels: the peripheral level (SD) with HC and district hospitals (DH); the intermediate level (SP) with regional hospitals; and the central level (DNHIS) with national and specialised hospitals. Routine data on diseases registered in consultations and laboratory (e.g. malaria, diarrhoea, schistosomiasis) are collected from each HC and reported to the SD, which reports in turn to the SP and the SP reports to the DNHIS. For hospitals, they report to the SD or to the SP or to the DNHIS, according to the level they belong to. Reporting is done monthly for more than 95% of diseases, including intestinal schistosomiasis.

### Data collection

In 2017, routine data on intestinal schistosomiasis were collected from the DNHIS. The data concerned all intestinal schistosomiasis cases as determined by DS and reported by the HC from each SD during the period 2011 to 2015. They were used for this study with the authorisation of the Ministry of Health. Data from hospitals were not collected to avoid duplicates as hospitals receive patients referred by HC.

### Data analysis

We described the number of reported cases based on DS detection per year in each SD over a period of 5 years, with a focus on 2014, where the DS-based incidence at HC level could be compared with the KK-based prevalence in school children.

We analysed temporal trends in intestinal schistosomiasis incident cases for each year in the 11 SD targeted by MDA campaigns (zone of intervention-ZI) with PZQ, and compared them with those in the 34 remaining SD (zone of non-intervention-ZNI), to allow for any general trend in intestinal schistosomiasis, not related to annual MDA by PZQ.

The number of intestinal schistosomiasis cases reported each year were put on a logarithmic scale. The slope should be linear if the incidence remained identical, despite the progressive growth of the population at risk (assumed to be identical for all SD, after the general census of 2008). The effect of MDA campaigns on the annual incidence of intestinal schistosomiasis should manifest itself as a significant change in the slope.

A mixed negative binomial regression was performed due to the overdispersion of the data [13, 14]. A random effect was added to take into account the existing correlation in the data per district over time. The trends of the number of intestinal schistosomiasis cases reported each year were compared between ZI and ZNI. The respective slopes *β*_i,_ i = 2012, 2013, 2014, 2015, were estimated to compare the trends in ZI and ZNI each year (2012, 2013, 2014 and 2015) to the reference year 2011. A second analysis was performed, for which only one slope *β* was estimated to study the overall trend during the period of 5 years (from 2011 to 2015). The slope estimation was given for both zones ZI and ZNI. Stata version 12 (Stata Corp. LP, College Station, USA) software was used to calculate the slopes with an α risk error of 5%.

## Results

The routine data obtained by DS showed a decreasing schistosomiasis incidence rate in the majority of the SD in the ZI, and a stable incidence rate in the majority of the SD in the ZNI. However, there were some exceptions in both zones. In the ZI, the incidence rate in the SD of Rumonge remained stable over time and in the ZNI, the incidence rates in some of the SD decreased, while they increased in others (Table 1).

**Table 1:** Number of intestinal schistosomiasis cases per sanitary district and per year as determined by direct smear at health centre level in Burundi

SD name	Zone	2011	2012	2013	2014	2015
Bubanza	ZNI	99	78	36	143	63
Rwibaga	ZNI	103	14	0	2	6
Bururi	ZNI	8	1	2	14	22
Matana	ZNI	32	9	21	19	20
Cankuzo	ZNI	10	1	0	2	0
Murore	ZNI	1	18	4	0	0
Mabayi	ZNI	64	74	38	30	53
Gitega	ZNI	27	17	24	55	31
Kibuye	ZNI	0	1	5	1	2
Mutaho	ZNI	13	2	3	6	1
Ryansoro	ZNI	4	0	3	4	2
Buhiga	ZNI	0	2	0	2	0
Nyabikere	ZNI	0	4	0	0	1
Kayanza	ZNI	6	6	8	7	69
Musema	ZNI	5	7	2	31	8
Gahombo	ZNI	30	25	18	6	15
Mukenke	ZNI	13	4	4	5	0
Vumbi	ZNI	27	26	26	26	1
Makamba	ZNI	238	267	249	270	195
Muramvya	ZNI	0	19	3	103	2
Kiganda	ZNI	0	5	6	3	15
Muyinga	ZNI	28	17	8	19	31
Gashoho	ZNI	38	9	1	0	0
Giteranyi	ZNI	0	22	152	7	12
Kibumbu	ZNI	10	2	40	4	19
Fota	ZNI	0	0	0	0	0
Ngozi	ZNI	5	1	0	32	0
Kiremba	ZNI	9	97	3	34	0
Buye	ZNI	0	0	0	0	0
Rutana	ZNI	12	30	15	5	2
Gihofi	ZNI	15	68	72	16	13
Butezi	ZNI	9	4	0	5	2
Kinyinya	ZNI	69	25	99	125	103
Ruyigi	ZNI	1	1	6	2	1
Mpanda	ZI	585	397	307	121	118
Zone Nord	ZI	493	483	215	118	50
Zone Centre	ZI	504	9	70	22	36
Zone Sud	ZI	145	24	23	11	39
Kabezi	ZI	115	47	56	174	82
Isale	ZI	210	156	71	346	53
Rumonge	ZI	189	136	374	213	351
Cibitoke	ZI	883	996	901	296	169
Kirundo	ZI	10	21	9	12	3
Busoni	ZI	23	23	103	15	4
Nyanza-Lac	ZI	247	315	255	263	149

*Abbreviations*: SD, sanitary district; ZI, zone of intervention, targeted for MDA with PZQ; ZNI, zone of non-intervention, not targeted for MDA with PZQ

**Fig. 1:**
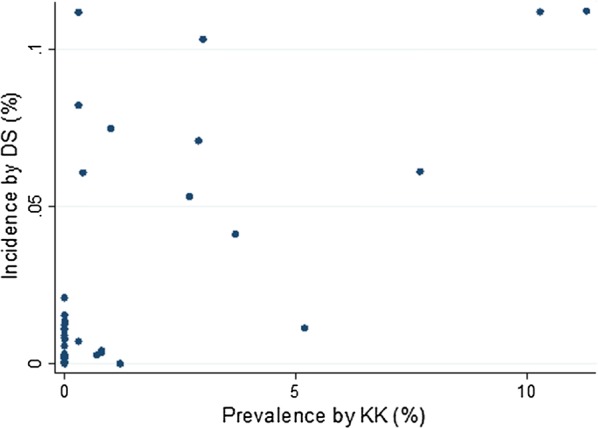
Prevalence (%) by kato-katz test in school children and incidence (%) by direct smear at health centre level in 2014, Burundi

We were able to compare the data of 2014 (Fig. 1) where both district-wide routine surveillance data and school-based mapping data were available. In most SD the low prevalence was confirmed by a low incidence rate. However, in some SD (e.g. Bururi, Gitega, Musema, Zone Centre) the prevalence rate was zero, while routine surveillance detected some cases. The two districts with the highest prevalences also showed the highest incidence rates, but relatively high incidence rates were also seen in SD with lower prevalences.

**Fig. 2:**
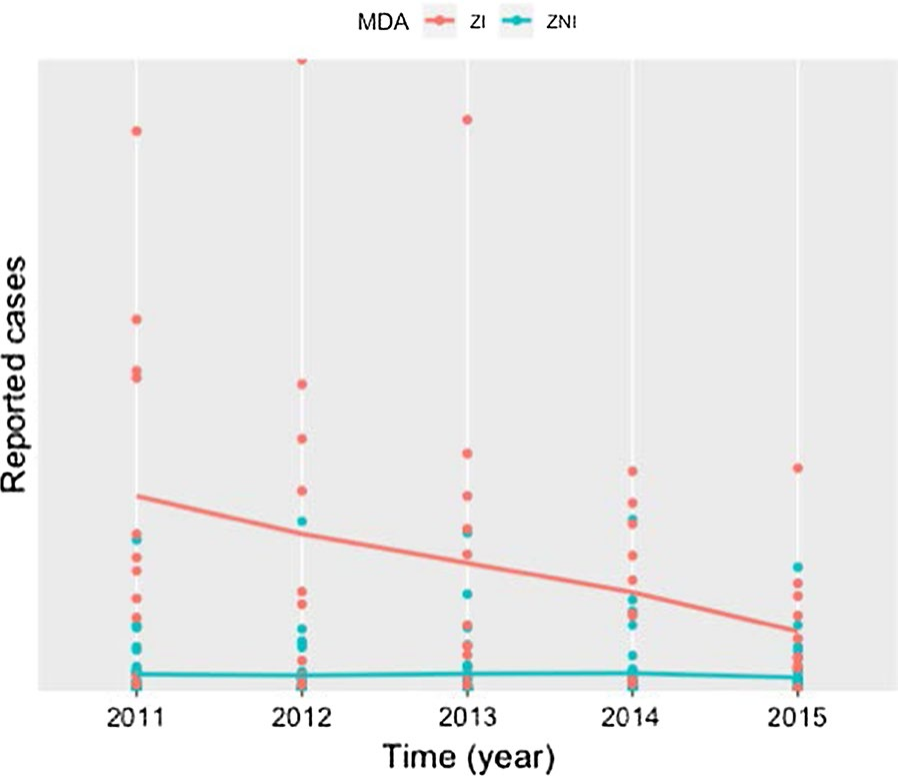
Temporal trends in schistosomiasis in the zone of intervention (ZI), targeted for MDA with PZQ and in the zone of non-intervention (ZNI), not targeted for MDA with PZQ

Table 2 shows the results of the temporal trends analysis. Both in the ZI and the ZNI, the incidence rate decreased, but this only reached significance in the ZI for 2014 (*β*_2014_ = − 0.826, *P* = 0.010) and 2015 (*β*_2015_ = − 1.294, *P* < 0.001). The overall trend over the five years period (2011 to 2015) showed a highly significant decrease in the ZI (*β* = − 0.286, *P* < 0.001), while no significant trend was observed in the ZNI (*β* = − 0.087, *P* = 0.219) (Fig. 2).

**Table 2:** Analysis of annual trends in intestinal schistosomiasis cases in zone in Burundi that was targeted for MDA with PZQ (ZI) and zone that was not (ZNI)

Year	Zone	SD (*N*)	*n*	β	95% CI	*P*-value
2011	ZI	11	3404	1	–	–
	ZNI	34	876	1	–	–
2012	ZI	11	2607	− 0.563	− 1.180–0.054	0.074
	ZNI	34	856	− 0.157	− 0.757–0.443	0.607
2013	ZI	11	2384	− 0.521	− 1.140–0.097	0.098
	ZNI	34	848	− 0.324	− 0.940–0.292	0.302
2014	ZI	11	1581	− 0.826	− 1.454–− 0.198	0.010*
	ZNI	34	978	− 0.055	− 0.663–0.553	0.859
2015	ZI	11	1054	− 1.294	− 1.908–− 0.680	< 0.001*
	ZNI	34	689	− 0.508	− 1.121–0.105	0.104
2011–2015	ZI	11	–	− 0.286	− 0.429–− 0.144	< 0.001*
	ZNI	34	–	− 0.087	− 0.227–0.052	0.219

*Abbreviations*: CI, confidence interval; SD, sanitary district; *n,* number of cases of intestinal schistosomiasis; N, number of sanitary districts; ZI, zone of intervention, targeted for MDA with PZQ; ZNI, zone of non-intervention, not targeted for MDA with PZQ

*P < 0.05

## Discussion

MDA campaigns with PZQ are recommended for the control of schistosomiasis [15–17] and constitute the current national strategy for schistosomiasis control in Burundi [18]. The KK test is still the primary diagnostic tool in monitoring the impact of national school-based deworming programmes on *S. mansoni* infection [15, 17], but it is not available in Burundi as a routine test at PHC level. The only test available for the diagnosis of intestinal schistosomiasis in Burundian HC is the DS [18]. We explored whether DS results that are routinely collected at HC level could give an indication of the impact of MDA with praziquantel on *S. mansoni* infection in Burundi as well. We did this by comparing temporal trends in intestinal schistosomiasis as determined by DS in HC of ZI with those in ZNI. A decreasing trend was observed in the ZI, but not in the ZNI. The decreasing trend in the ZI was in line with the decline in schistosomiasis prevalence from 12.7% in 2007 to 2.2% in 2014 as observed with the gold standard KK test [15] performed in the epidemiological surveys in sentinel sites and in the national mapping [10]. Moreover, comparison of routine surveillance data with school-based mapping data per SD in 2014 showed that for most SD low prevalences based on KK were confirmed by low incidence rates by DS, and that in some districts where the prevalence with KK was zero, routine surveillance still detected some cases.

In contemporary Burundi, schistosomiasis transmission is low to moderate, the health care system is weak, and the (national) resources for health care limited. Furthermore, schistosomiasis diagnosis through KK and treatment of positive cases with PZQ is absent in the primary health care setting [18]. Despite these limitations, the current passive routine surveillance system of Burundi appears still be able to monitor the evolution of schistosomiasis in the ZI as well as in the ZNI. Both are equally important in the framework of disease elimination.

Our data hold promise for the use of routine diagnostic data collected at HC level as a cost-effective and sustainable strategy to monitor the impact of MDA campaigns and other schistosomiasis control interventions in Burundi. However, it should be borne in mind that DS, but also KK, have a reduced sensitivity especially in areas of low endemicity, such as Burundi [10]. Highly sensitive diagnostic tools to detect light-intensity infections are pivotal for monitoring progress from control towards elimination [19–21]. Several studies have now documented the lateral flow immunochromatographic point-of-care (POC) test detecting *Schistosoma* circulating cathodic antigen (CCA) in urine as a valuable alternative to the KK for the diagnosis of *S. mansoni* [22]. It is rapid, user-friendly, and considerably more sensitive than KK especially in areas of low prevalence [23]. While the POC-CCA assay has already shown its value as a mapping and monitoring in national control programmes [10, 22], its suitability for integration into the PHC system as a routine test for case management and surveillance/monitoring of intestinal schistosomiasis still needs to be investigated in more detail. Burundi is low endemic for intestinal schistosomiasis and currently aiming for elimination [10]. This, in combination with a relatively well-functioning NHIS system, would provide an ideal setting for such a study.

## Conclusions

The results of this preliminary study suggest that routine surveillance at HC level may be able to monitor the impact of MDA with PZQ on intestinal schistosomiasis in Burundi. However, more sensitive routine diagnostic tests, such as the POC-CCA assay are desirable when moving from control to elimination of schistosomiasis. Integration of adequate diagnosis and treatment into the routine activities of primary health care facilities is essential for intestinal schistosomiasis to be controlled and eventually eliminated in Burundi.

## Data Availability

Data supporting the conclusions of this article are included within the article. The datasets generated and analysed during the present study are available from the corresponding author upon reasonable request.

## Abbreviations

CCA: circulating cathodic antigen
DH: district hospital
DNHIS: Directorate of National Health Information System
DS: direct smear
HC: health centre
HF: health facility
KK: kato-katz
MDA: mass drug administration
NHIS: National Health Information System
NTD: neglected tropical diseases
PHC: primary health care
POC-CCA: point-of-care circulating cathodic antigen
PZQ: praziquantel
SCI: Schistosomiasis Control Initiative
SD: sanitary district
SP: sanitary province
WHO: World Health Organization
ZI: zone of intervention
ZNI: zone of non-intervention
